# Developing a Live Probiotic Vaccine Based on the *Enterococcus faecium* L3 Strain Expressing Influenza Neuraminidase

**DOI:** 10.3390/microorganisms9122446

**Published:** 2021-11-27

**Authors:** Yulia Desheva, Galina Leontieva, Tatiana Kramskaya, Tatiana Gupalova, Igor Losev, Eugenia Kuleshevich, Elena Bormotova, Olga Kopteva, Polina Kudar, Alexander Suvorov

**Affiliations:** Scientific and Educational Center “Molecular Bases of Interaction of Microorganisms and Human” of the World-Class Research Center “Center for Personalized Medicine” FSBSI “IEM”, 197376 Saint Petersburg, Russia; galeonte@yandex.ru (G.L.); tatyana.kramskaya@gmail.com (T.K.); tvgupalova@rambler.ru (T.G.); iemlosev@gmail.com (I.L.); k-zh-v@mail.ru (E.K.); bormotovae@rambler.ru (E.B.); olga.s.kopteva@yandex.ru (O.K.); polina6226@mail.ru (P.K.); alexander_suvorov1@hotmail.com (A.S.)

**Keywords:** *E. faecium* L3, probiotic microorganism, live mucosal vaccine, influenza neuraminidase

## Abstract

Probiotic microorganisms are currently considered as a promising platform for the development of recombinant vaccines expressing foreign antigens. In this study, we generated and evaluated the live mucosal recombinant vaccine by integrating genes encoding influenza virus neuraminidase (NA) of the N2 subtype into the DNA of the probiotic strain *Enterococcus faecium* L3 (L3). We confirmed NA expression in the pili of L3 using immune electron microscopy. Mice were fed with a probiotic vaccine containing the NA gene (L3-NA) or pure L3. Oral administration of L3-NA caused detectable increase in virus-specific serum IgG and local IgA after the third feeding. Immunization with L3-NA increased the survival rate by 34% when the mice were infected using A(H1N1)pdm09 influenza virus after the third feeding. After *S. pneumoniae* post-influenza infection, the L3-NA-immunized mice were 50% more protected from lethality in comparison with L3-fed mice. Thus, a live probiotic vaccine candidate based on L3 induced the formation of systemic and local immunity and provide partial protection against complicated influenza.

## 1. Introduction

Probiotics are used as vectors, some of which were successfully introduced with plasmid constructs that provide the expression of antigens of pathogenic bacteria [1]. One of the most promising approaches to the creation of mucosal vaccines is the use of probiotic vectors carrying viral components. This approach promotes both the formation of humoral immunity, which has broad specificity for influenza virus strains, and the enhancement of the nonspecific activation of the immune system due to the immunomodulatory properties of the probiotic [2]. In addition, like other live vaccines, these probiotic preparations promote the formation of the cellular component of adaptive immunity.

The main advantage of live mucosal vaccines is the activations of all components of the immune system which induce a balanced, strong immune response. Live intranasal influenza vaccines (LAIVs), which are especially effective in vaccinating children, are widely used [3,4], as a rule, once a year. Oral administration of a probiotic vaccine may help to provide immunization among the population groups for whom vaccination is not allowed, as well as to practice frequent and repeated immunization, if necessary, such as against seasonal respiratory infections.

In this study, the well-studied probiotic strain *Enterococcus faecium* L3 (L3), which has a number of unique biological properties [5,6,7,8], was used as a recipient bacterium. The strain *E. faecium* L3 has a pronounced antagonistic activity against Gram-positive and Gram-negative bacteria, the ability to restore intestinal microbiota against the background of dysbiosis conditions [6], as well as an immunomodulatory effect on the host organism [7]. *E. faecium* L3 synthesize multiple bacteriocins having inhibitory activities against Gram-positive and Gram-negative pathogens, which have not only antibacterial action, but also show antiviral properties in vitro [5,6].

In the *E. faecium* L3 strain, like other Gram-positive bacteria, pili were found, which are fimbria 0.3–3 μm in length and 2–10 nm in diameter [9]. These long, protein-like polymers stretching on the surface of bacteria appear as subunits of the pilin protein linked by a covalent bond. They play a significant role in adhesion and host colonization. Pili are highly immunogenic structures that are under the selective pressure of the host immune responses [10]. The principle of *E. faecium L3* pili modification with vaccine antigens is an attractive approach for creating effective live vaccines due to exposure of the target antigen on the surface of *Enterococcus*. 

The objective of the study was to create a live vaccine based on a biologically active strain of *E. faecium* L3 by including a fragment of the NA gene of the influenza A17/duck/Potsdam/86/92 (H5N2) virus in the structure of its pili. The vaccine strain A/17/duck/Potsdam/86/92 (H5N2) was obtained using classical genetic reassortment of non-pathogenic avian influenza virus A/H5N2 and master donor strain (MDS) A/Leningrad/134/17/57(H2N2), and inherited N2 from A/H2N2 MDS [11]. Currently, the greatest attention in the development of influenza vaccines is focused on hemagglutinin (HA), although the high variability of influenza viruses reduces the effectiveness of vaccination when the infecting virus differs from the vaccine strain [12]. The HA of seasonal influenza viruses is under significant immune pressure on the human population, and therefore, its evolutionary rate is significantly higher than that of NA [13]. The use of influenza neuraminidase (NA) as a vaccine antigen is a promising approach due to broad cross-reactive the broadly protective effect of NA antibodies [14].

The contribution of anti-NA antibodies for protection against seasonal influenza viruses has been actively discussed in recent years in connection with the urgent need to improve the effectiveness of seasonal influenza vaccines [15,16,17]. Antibodies against NA are capable of providing heterosubtypic protection [18], which is especially important in view of the potential threat to humans from avian influenza viruses, which can cross the interspecies barrier and are considered possible causative agents of a future influenza pandemic [19].

## 2. Materials and Methods

### 2.1. Molecular Analysis

Multiple alignments and the identity analysis of the NA amino acid sequences from various influenza viruses were carried out using the GeneDoc program, version 2.6.002 (https://genedoc.software.informer.com/2.6/). The amino acid sequences of the NA proteins were obtained from the GenBank database (www.ncbi.nlm.nih.gov). The names and types of influenza viruses used for the analysis of NA nucleotide sequences used in this study are presented in Table 1.

A three-dimensional model of the NA insertion (AA 123–331) was created using Cn3D software [20]. To search for conserved NA epitopes, we used the AAPPred [21], ABCPred [22], BepiPred [23], COBEpro [24], ElliPro [25], and Epitopia [26] software with the subsequent generalization of the results obtained from each. The data obtained based on epitopes of the A/Leningrad/134/17/57 (H2N2) influenza virus were chosen according to the following conditions: the NA region should have been predicted as a linear epitope by at least 3 methods, and the predicted region must match the conserved NA region of all influenza A viruses used in the study.

### 2.2. Obtaining a Chimeric Protein from E. faecium L3 d2 Gene and a Fragment of the NA Gene

DNA fragments corresponding to the NA gene coding 208 AA (123–331) were embedded into recombinant DNA plasmid pentF-pspf, obtained by inserting a total DNA fragment consisting of two separate fragments of the probiotic *E. faecium* L3 gene and a chimeric pneumococcal protein (PSPF) gene fragment, described in [27]. The oligonucleotide primers used for the reaction are listed in Table 2.

RNA of the A/17/duck/Potsdam/86/92 (H5N2) influenza virus was isolated from the virus-containing allantoic fluid using the Viral RNA Mini Kit (Qiagen, Hilden, Germany). DNA on the viral RNA template was obtained using a single-step reverse transcriptase polymerase chain reaction (PCR) with primers EV and FV using the OneStep RT-PCR Kit (Qiagen, Hilden, Germany). The resulting PCR product was excised from an agarose gel and digested with NdEI and EcoRI. Cloning of the restriction fragment after amplification of the DNA fragment encoding the NA fragment was carried out using the upper fragment. The plasmid pentF-pspf encoding pneumococcal surface protein F (PspF) within the d2 gene from *E. faecium* L3-encoding pili protein [27] was hydrolyzed by the NdeI and EcoRI enzymes to remove the pspf gene, and the remaining fragment was used for the subsequent cloning of viral epitope.

Restriction products were separated by electrophoresis in 1% agarose gel. Restricted DNA was isolated from agarose using a QIAquick Gel Extraction Kit (Qiagen, Hilden, Germany), ligated and transformed into *E. coli* DH5α system. The selection medium contained 500 μg/mL erythromycin (er). DNA was isolated from the obtained a number of clones after transformation, which were tested in the PCR reaction with primers EV and FV and with specially designed primers SeqF and SeqR, where 8 transformants were positive. From two of transformants, plasmids were isolated that contained the expected insert. The presence of the insert was confirmed by hydrolysis of the plasmid with NdEI and EcoRI. Thus, as a result of cloning, the pentF-na plasmid with the expected insert and erythromycin resistance gene was obtained.

The *Enterococcus faecium* L3 culture was transformed by the integrated plasmids using electroporation procedure as described earlier [27].

One of the transformants was selected and designated as the NA-positive clone (L3-NA). Amplification of DNA isolated from this clone was carried out using B1 and FV primers and B1 and Seq R primers for sequencing. Sequencing of DNA isolated from the NA+ clone was carried out with primers corresponding to the NA gene sequence (primer Seq R) and the *Enterococcus* chromosomal DNA sequence (primer B1) to confirm the integration of pentF-na plasmid DNA into *Enterococcus* chromosomal DNA (Supplementary Data S1).

### 2.3. Real-Time Reverse Transcriptase PCR (rRT-PCR)

To confirm the expression of the inserted NA gene in bacterial DNA, we studied the expression of mRNA using real-time reverse transcriptase PCR (rRT-PCR) with NA-specific primers. Bacteria were grown in THB (Todd Hewitt Broth (Condalab, Madrid, Spain)) medium at 37 °C for 18 h. *E. faecium* L3-NA was cultivated with 5 μg/mL of erythromycin. For mRNA analysis, *E. faecium* L3 was collected at the logarithmic phase, when OD600 reached a value of 0.8–0.9, which corresponded to 3–5 × 10^8^ CFU/mL bacteria. Bacteria were washed three times in PBS by centrifugation at 3500 rpm for 20 min and suspended in PBS. Then, 10× concentrate was used for m RNA analysis. Isolation of total RNA was carried out using the GeneJET RNA Purification Kit (Thermo Scientific, Waltham, MA, USA) according to the manufacturer’s instructions. The isolated RNA was treated with 1 U/µL DNase (Invitrogen, Waltham, MA, USA), after which one-step rRT-PCR was performed in on a SFX96 thermocycler (Biorad, Hercules, CA, USA) using HS-qPCR SYBR Blue master-mix (Biolabmix, Novosibirsk, Russia). As a normalizing gene, we used D-alanine-D-alanine ligase gene of *E. faecium* L3 with the following primers: F-TTGAGGCAGACCAGATTGACG, R-TATGACAGCGACTCCGATTCC. Primers corresponding to the NA gene sequence were EV and FV (Table 1).

### 2.4. Study of Expression of NA Protein on the Surface of E. faecium L3 Using ELISA 

The modified and unmodified 24-h *Enterococcus* culture was sorbed onto 96-well ELISA plates (Thermo Scientific, Waltham, MA, USA) for a day. For this, the bacterial suspension was grown as mentioned above and washed 3 times in PBS, after which the protein concentration was determined by the Lowry method [28] and diluted to a final protein concentration of 2 μg/mL. After that, serial dilutions of human blood sera with known titers of antibodies to NA were introduced into the wells of the plate. Thereafter, ELISA was performed as described earlier [11] using goat antibodies to human IgG (Sigma-Aldrich, St. Louis, MO, USA) as a conjugate. The presence of NA-inhibiting (NI) antibodies in human sera ≥ 1:160 was confirmed in the enzyme-linked lectin assay (ELLA) test as previously described [29]. The titer of serum NI antibodies was determined as the reciprocal dilution of the sample with 50% inhibition of NA activity.

### 2.5. Immunoelectron Microscopy to Study the Structure of E. faecium L3 Pili with Expression of Viral Proteins

Bacteria were grown in Lysogeny broth medium (VWR Life Science Products Amresco, Solon, OH, USA) at 37 °C for 18 h. *E. faecium* L3-NA was cultivated with 5 μg/mL of erythromycin. Bacteria were washed three times in PBS by centrifugation at 3500 rpm for 20 min and suspended in 0.1 M NaCl. Then, 10× concentrate was used for microscopy. Immuno-gold labeling was performed using human polyclonal antibodies specific for NA as primary antibodies and goat IgG conjugated to 18 nm gold particles (1 mg/mL; Jackson ImmunoResearch Laboratories, West Grove, PA, USA). The grids for transmission electron microscopy (G4901-1VL # 3110 SPI-Grids, 300 mesh 83 µm pitch, Sigma-Aldrich, St. Louis, MO, USA) were coated with trinitrocellulose in the mix ethanol and diethyl ether in a ratio of 1:7. The bacterial culture samples were applied to the grid by the method for liquid suspensions (“drop on the grid”) followed by incubation for 2 min at room temperature. Then, the grids were fixed for 1 min in (2.5% paraformaldehyde in PBS). Blocking was performed with 0.1% gelatin in PBS for 1 h. Primary antibodies were diluted in 2% BSA/PBS, incubation lasted for 1 h. Secondary antibodies were diluted 1:20 in 2% BSA/PBS, and the samples were incubated for 1 h. For contrasting, 1% uranyl acetate was used in a drop of 20 μL (20 s). To compact the resulting films, they were covered with a layer of nanocarbon. Electron microscopy was performed on a JEM-2100 transmission electron microscope (JEOL, Tokyo, Japan). Photos were taken with digital cameras: bottom port—Gatan Ultrascan 4000 16 Mpix, 4 × 4 K, 16-bit; side port—Gatan Erlangshen 500 1.4 Mpix, 1.3 × 1 K, 12-bit, 15 fps (Gatan, Pleasanton, CA, USA).

### 2.6. Oral Immunization of Mice with Live Probiotic Vaccine

The CBA mice (*n* = 90) or Balb/c mice (*n* = 90) were randomly distributed in three groups (*n* = 30 per group). The CBA mice were used as one of the most famous early inbred lines including CBA and BALB/c [30]. Each group of mice was orally immunized using a mouse feeding needle. A total volume of 500 µL of a probiotic vaccine containing an insert of NA gene (L3-NA) or pure *E. faecium* L3 strain (L3) at a dose of 5 × 10^9^ colony forming units (CFU) per mouse was administered on day 1, 14 or 28 from the start of experiment. Mice in control groups were fed by PBS. Blood serum and nasal samples were collected from 10 mice per group to determine NA-specific ELISA antibodies. Saliva/nasal samples were collected from 10 mice per group after intraperitoneal administration of 0.1 mL of a 0.5% pilocarpine solution into the tubes containing 0.001 M of serine protease inhibitor phenylmethylsulfonyl fluoride (PMSF).

### 2.7. Evaluation of L3-NA Persistence in the Gastrointestinal Tract of Vaccinated Mice

To study the duration of bacterial persistence in the intestine of animals, mouse feces were collected daily after the third vaccination and frozen at −80 °C. L3-NA was qualitatively determined by spreading feces homogenate onto a selective agar surface. *Enterococcal* (azide) agar containing erythromycin at a concentration of 5 μg/mL was used as the elective medium. The feces samples were homogenized with a bead shaker μT-12 (TAITEC, Saitama, Japan) at 3200 rpm for 30 s. Suspensions used contained final feces amounts of 100 μg/mL. Then, 10 μL of the supernatant was applied to the agar surface and the plates were incubated for 24 h at 37 °C for the counting of colonies.

### 2.8. Ethic Statement

All of the animal experiments were carried out under the guidelines of the Rules of Laboratory Practice of the Ministry of Health of the Russian Federation N° 708. The study was approved by the Local Ethics Committee for Animal Care and Use at the Institute of Experimental Medicine, Saint-Petersburg, Russia, with the protocol number 1/20 from 25 February 2020.

### 2.9. Determination of NA-Specific IgG and IgA

Serum and local IgG and IgA levels were determined using ELISA in 96-well ELISA plates (Sarstedt, Nümbrecht, Germany) coated with A/Leningrad/134/17/57(H2N2), A/California/07/09(H1N1)pdm09 or A/South Africa/3626/13(H1N1)pdm09 (20 hemagglutination units, HAU) per 0.1 mL of the whole virus purified on sucrose gradient) overnight at 4 °C, as previously described [11]. The starting dilution for IgG was 1:40, and for IgA, 1:4 or 1:10. The endpoint ELISA titers were expressed as the highest dilution that yielded an optical density at 450 nm (OD_450_) greater than the mean OD_450_ plus 3 standard deviations of negative control wells.

### 2.10. Study of Protection against Influenza Infection after Vaccination with Live Probiotic Vaccine

At 10 days after the end of the third immunization, the mice were intranasally infected with five 50% mouse lethal doses (MLD_50_) of A/South Africa/3626/13(H1N1)pdm09 influenza virus. A group of immunized mice were additionally infected with *S. pneumoniae* clinical isolates serotype 3 strain 73 (5 × 10^4^ CFU) 24 h after influenza infection. *S. pneumoniae* were cultured in anaerobic conditions at 37 °C for 18 h in THB medium with 20% horse serum (Difco, Carrickmore, UK). The Columbia agar with 5% defibrinated sheep blood and 10% horse serum was used as a solid medium for cultivation and counting of the bacterial number. The lungs were homogenized in PBS using a Retsch MM-400 ball vibratory mill. Serial 10-fold dilutions of homogenates were made in PBS and aliquots of the dilutions were plated on Columbia agar. Plates were incubated at 37 °C in 5% CO_2_ for 14–16 h before the colonies were counted under a microscope. The bacterial burden in colony forming units (CFU) was calculated and expressed as log10. To determine the lung viral titer, the lung samples were homogenized in PBS containing 100 U/mL penicillin and 100 μg/mL streptomycin and centrifuged for 10 min at 6000× *g*. The viral titers were calculated as 50% embryonic infectious dose (EID_50_) in chicken embryos (CE) using hemagglutination as the endpoint as described previously [11]. 

### 2.11. Statistics

Statistical processing of the results was carried out using the GraphPad software (San Diego, CA, USA). For statistical analysis, the antibody titers were expressed as log2 of the inversed final dilution. Comparisons of two independent groups was performed using the nonparametric Mann–Whitney test. The log-rank (Mantel–Cox test) was used to compare the survival distributions. The *p* value < 0.05 was considered to be statistically significant.

## 3. Results

### 3.1. Molecular Analysis of NA Insertion

**Figure 1:**
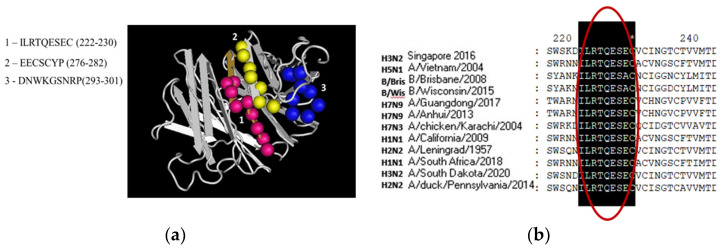
Molecular structure of NA insertion. (**a**) Three-dimensional model of the NA insertion (AA 123–331) built on the basis of the NA model of the 1957 pandemic influenza H2N2 virus; (**b**) Multiple alignment of influenza A and B viruses NA.

To search for conserved NA epitopes, a number of amino acid sequences of various influenza A viruses were selected. The currently existing prediction algorithms are not capable of predicting linear B cell epitopes with high accuracy and sensitivity. Therefore, to achieve greater accuracy, several methods were used with the subsequent generalization of the results obtained from each of them. Figure 1a shows epitopes that are predicted using several methods. The putative insert of influenza neuraminidase of N2 subtype contained a number of B cell epitopes, including the epitope 222–230 described as common to influenza A viruses [31] (Figure 1a). This sequence is also present in the NA structure of influenza B viruses (Figure 1b). 

### 3.2. Generation of the Live Probiotic Influenza Vaccine Candidate

**Figure 2:**
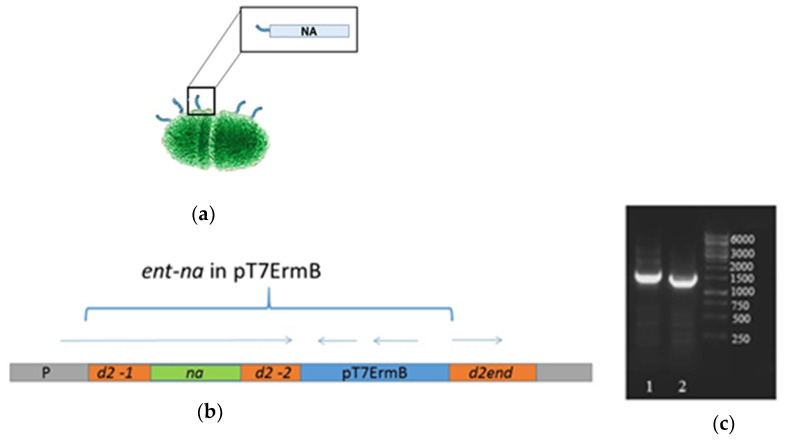
Generation of the live probiotic influenza vaccine candidate. (**a**) Schematic presentation of probiotic *E. faecium* vector; (**b**) Integration scheme of the plasmid pT7ermB with the ent-na into the chromosome of the strain *E. faecium* L3. P is the promoter of the gene d2; d2-1 is a region of the d2 gene encoding for the N-terminal part of D2 protein (209 amino acids—GenBank: HAQ0044242.1); d2-2 is a region of the d2 gene encoding for central and major part of C terminus portion of D2 protein (284–393 amino acids); na is DNA encoding neuraminidase; d2-end is the end of the d2 gene encoding for the C terminus of D2 protein (394–405 amino acids); pT7 ErmB is the integrative plasmid. Arrows correspond to the open reading frames in the integrated element. The entire integrated element ent-na with plasmid pT7ErmB is shown in brackets; (**c**) Agarose gel electrophoresis of the amplified bacterial gene d2 containing inserts of influenza virus gene fragments: 1, L3-NA; 2, L3.

A live probiotic influenza vaccine candidate was generated by standard gene engineering approaches. Chimeric DNA construction containing the NA element was inserted into the d2 Enterococcus gene, resulting in the exposure of the influenza virus antigen on the surface of bacteria as a part of their pili (Figure 2a,b). Amplification of the d2 bacterial gene fragment demonstrated that the desired influenza gene was successfully inserted into the bacterial genome (Figure 2c).

### 3.3. Expression Levels of the NA mRNA in the Bacteria

**Figure 3:**
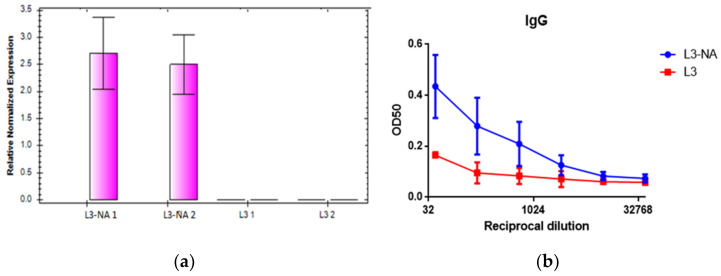
Study of NA expression in a modified *Enterococcus faecium* L3 culture. (**a**) The mRNA expression level of NA gene in a culture of bacteria. Data from two independent experiments are presented, samples are tested in duplicates: L3-NA-1 and L3-NA-2 (L3-1, L3-2) represent “experiment 1” and “experiment 2”, respectively. For normalized expression (∆∆C(t)), the gene of *Enterococcus faecium* L3 D-alanine-D-alanine ligase was used as a housekeeping gene, pure L3 was selected as a control sample; (**b**) Binding curves of patient’s sera (*n* = 5) containing antibodies to neuraminidase N2 subtype with *E. faecium* L3 cultures adsorbed on the surface of 96-well panels.

Estimation of mRNA expression after NA gene fragment integration into the genome of *E. faecium* L3 was performed using rRT-RCR analysis. When studied with oligonucleotide primers specific to the NA, the mRNA expression showed a 2.5–3-fold increase in NA-modified culture of *Enterococcus* (Figure 3a). At the same time, amplification did not occur when total RNA was isolated from a pure L3 culture.

In ELISA, it was shown that the serum containing NA antibodies to N2 in titers ≥1:160 bound to a culture of *E. faecium* L3 expressing NA (Figure 3b). A certain level of binding of NA-antibody containing sera with pure L3 was obtained; we explain this as nonspecific binding to similar proteins of enterococcus [32].

### 3.4. Immunoelectron Microscopy

To determine whether NA protein expressed on the pili of *Enterococcus faecium* L3, L3-NA was incubated with human polyclonal sera as a source of primary anti-NA antibody, and was exposed by anti-human IgG conjugated to 18 nm gold particles. A 1:100 dilution of primary antibodies was chosen, at which the difference between the binding of antibody-containing serum with pure L3 and L3-NA was most visible (Figure 3b).

**Figure 4:**
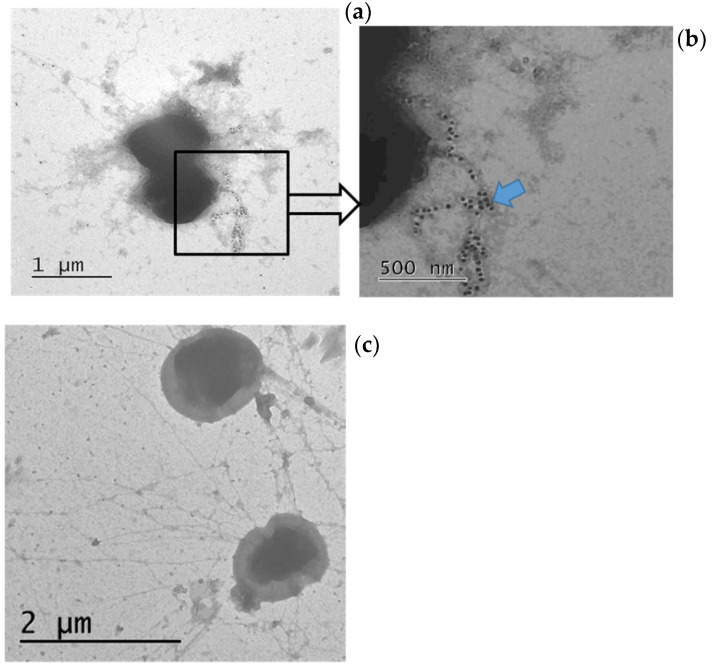
The structure of *E. Faecium* L3 pili with expression of NA. (**a**) *Enterococcus* with NA expression treated with primary antibodies to NA and secondary gold-conjugated goat anti-human IgG, 60,000× magnification; (**b**) The pili of *Enterococcus* with NA expression, 90,000× magnification; the blue arrow marks gold-conjugated goat anti-human IgG; (**c**) Pure *Enterococcus* treated with primary antibodies to NA and secondary gold-conjugated goat anti-human IgG, 60,000× magnification.

Our electron microscopy results showed that gold-conjugated antibodies are distributed along the entire length of the piles of *Enterococcus* L3 modified by NA (Figure 4a,b). When unmodified enterococcus L3 was treated in the same way, a chaotic arrangement of separately lying gold particles was observed (Figure 4c).

### 3.5. Bacterial Persistence in the Intestines of CBA Mice

**Figure 5:**
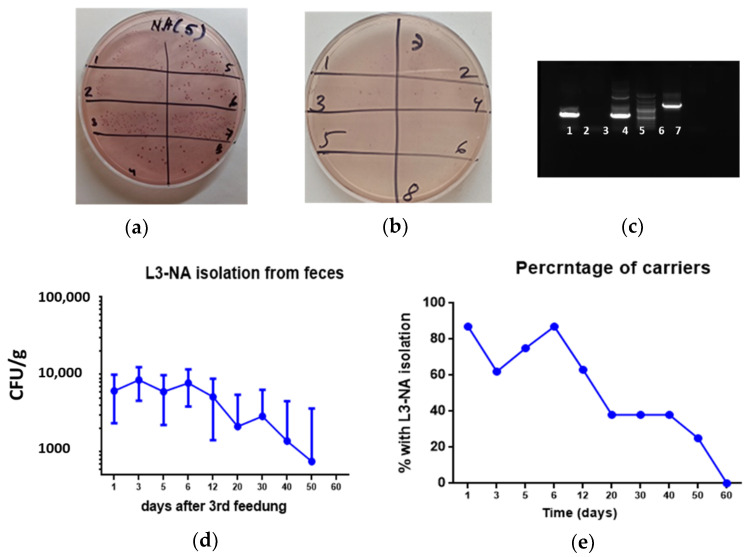
Bacterial persistence in the intestines of L3-NA immunized animals (*n* = 8). (**a**,**b**) Colonies of enterococcus on azide agar on day 5 and 60 after the 3rd immunization respectively; (**c**) NA identification by PCR using EV и FV primers (EV: 1, PCR product using template DNA from L3-NA colony (azide er+ agar); 2, PCR product using template DNA from L3 colony (azide er- agar); 3, PCR product using plasmid pentF-NA-1 template DNA; 4, 100 bp ladder DNA marker (100–3000 bp); 5, PCR product using template DNA from L3-NA colony (azide er+ agar); 6, PCR product using template DNA from L3 colony (azide agar er-) and B1 и FV as primer sequences; 7, PCR product without added DNA; (**d**) L3-NA isolation from stool samples; (**e**) Percent of mice with L3-NA isolation.

Stool samples were collected for 2 months after the third feeding. Enterococcal (azide) agar inhibits the growth of Gram-negative bacteria and does not prevent the development of *Enterococcus*. On enterococcal agar, L3-NA colonies are pink. Erythromycin in agar inhibits the growth of normal mouse enterococcal flora and does not prevent the development of L3-NA. Thus, the presence of L3-NA in the feces of mice can be judged by the development of pink colonies on a selective medium (Figure 5a,b).

It was shown that on day 3 after the end of feeding, L3-NA was not released in two out of eight mice (Figure 5a). Two months after immunization, no L3-NA was isolated in any mouse, which was confirmed by PCR tests carried out as shown in Figure 4c. Thus, it was shown that although L3-NA reproduced in the intestines of immunized animals, providing presentation of the NA, persistence was observed for no more than 2 months. The number of L3-NA isolated from the stool of vaccinated animals decreased within 2 months after the last immunization, as did the percentage of animals in which it was possible to detect L3-NA (Figure 5d,e).

### 3.6. Immunogenicity in CBA Mice

**Figure 6:**
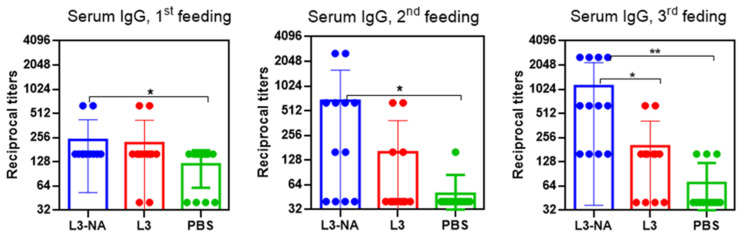
Immunogenicity in CBA mice estimated as serum IgG levels to A/Leningrad/134/17/57(H2N2) influenza virus (*n* = 10−12). Dots represent individual antibody titers in mice. * *p* < 0.05, ** *p* < 0.01.

The results of the study of immunogenicity after the first, second and third feedings with L3-NA are shown in Figure 6.

It was shown that after the first immunization, the titers of antibodies to the A/H2N2 influenza virus significantly exceeded those in the PBS group, but did not differ significantly from those in the group of mice receiving pure L3. Only after the third feeding serum did IgG levels significantly exceed those in the group of mice treated with pure L3. Therefore, further data of immunogenicity were determined after the third immunization.

**Figure 7:**
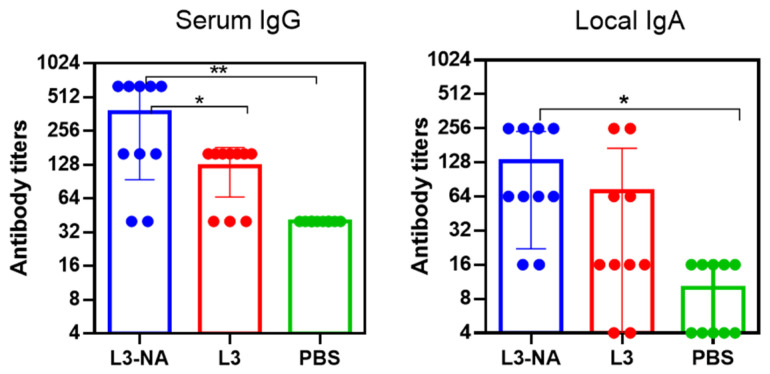
Antibody levels to A/California/07/09(H1N1)pdm09 influenza virus in CBA mice 14 days after the third feeding (*n* = 10). * *p* < 0.05, ** *p* < 0.01.

Oral administration of L3-NA to CBA mice caused an increase in virus-specific serum IgG as well as local IgA in nasal washes against antigenically distinct A/California/07/09(H1N1)pdm09 influenza virus after the third feeding (Figure 7). Determination of antibodies to heterologous N1 NA was performed since we planned to carry out challenge using a pandemic influenza virus A/H1N1pdm 09.

When the mice were fed by pure L3, some level of local IgA reacted with the whole influenza virus A/H1N1 was identified, but the antibody levels did not differ significantly from those in the group of mice immunized with PBS.

Thus, it was shown that oral immunization of CBA mice with a probiotic strain containing neuraminidase of N2 subtype elicited serum antibody response not only against homologous A/H2N2 but also against heterologous A/H1N1pdm influenza virus.

### 3.7. Protection against Lethal Influenza Infection

**Figure 8:**
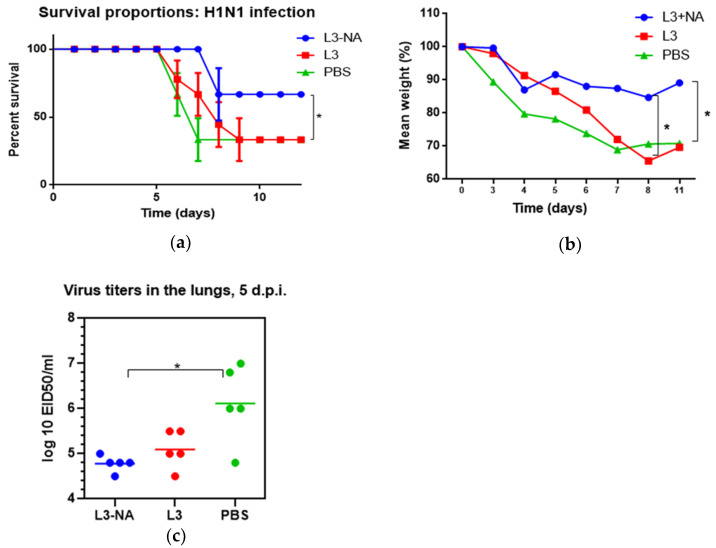
Protection against A/South Africa/3626/13(H1N1)pdm09 influenza infection; (**a**) survival proportions (*n* = 9); (**b**) body weigh dynamics (*n* = 12); (**c**) infectious virus isolation from the lungs (*n* = 5). * *p* < 0.05.

In a model of intranasal lethal influenza infection of mice, it was shown that three doses of mucosal probiotic vaccine L3-NA provided 67% protection against infection with 5 LD50 of A/South Africa/3626/13(H1N1)pdm09 influenza virus (Figure 8).

While 33% of the animals survived in the group of mice receiving pure L3 or PBS, 67% of the animals receiving the vaccine survived (Figure 8a). Mice immunized with L3-NA lost significantly less weight after A/H1N1pdm influenza infection compared to the groups receiving pure L3 or PBS (Figure 8b). The administration of L3-NA to mice also led to a decrease in the infectious virus in the lungs compared with the PBS group (Figure 8c). The positive effect of pure L3 on the course of A/H1N1pdm influenza infection consisted of delayed mortality and some decrease in the infectious virus in the lungs, which, however, was not statistically significant (Figure 8b,c).

### 3.8. Immunogenicity and Protection against Virus-Bacterial Infection in Balb/c Mice

**Figure 9:**
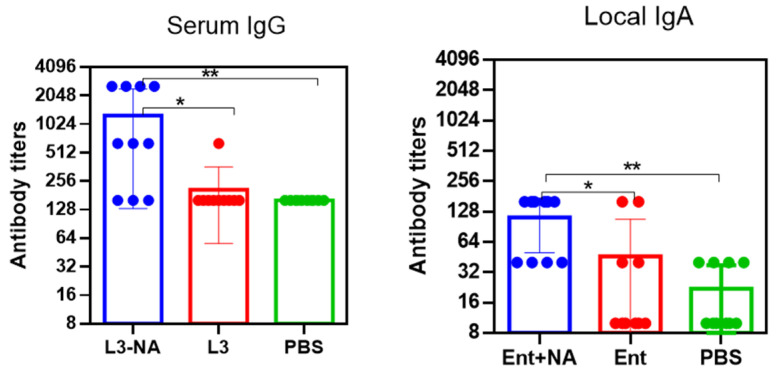
Immunogenicity in Balb/c mice 14 days after third feeding (*n* = 10). Serum IgG and local IgA to whole purified A/California/07/09(H1N1)pdm09 influenza virus (*n* = 10). * *p* < 0.05, ** *p* < 0.01.

Administration of L3-NA to Balb/c mice caused an increase in serum IgG and nasal IgA after the third feeding (Figure 9). 

The response of antibodies in CBA and BALB/c mice did not differ in magnitude. When assessing local immunity in Balb/c mice, statistically significant differences were observed between the L3-NA and L3 groups, in contrast to CBA mice. This suggests that the assessment of local antibodies in the study of such vaccine strains may better perform in Balb/c mice.

To study the protection against post-influenza bacterial infection, we chose the scheme when mice were infected with *S. pneumoniae* 24 h after primary influenza infection. This regimen was based on the fact that the nasopharyngeal carriage of *S. pneumoniae* can reach 53% in children [33], and primary influenza infection may activate existing latent bacterial infections.

**Figure 10:**
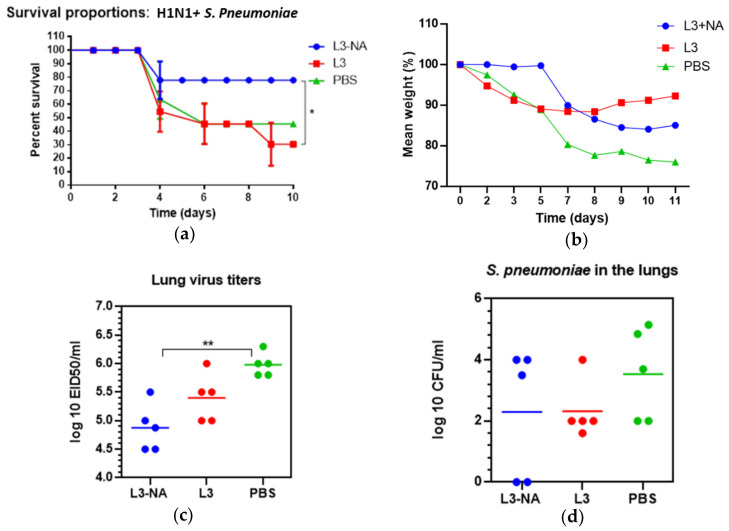
Protection against A/South Africa/3626/13(H1N1)pdm09 influenza infection followed by *S. pneumoniae* super-infection. On day 10, after the end of the third feeding, the mice were intranasally infected with one MLD_50_ of A/South Africa/3626/13(H1N1)pdm09 influenza virus; infection with *S. pneumoniae* serotype 3 (5 × 10^4^ CFU) was performed 24 h after primary influenza infection. (**a**) Survival proportions (*n* = 10). Log-rank (Mantel–Cox) test, * *p* < 0.05 compared to L3 group; (**b**) body weigh dynamics; (**c**) infectious virus isolation from the lungs on day 5 following primary influenza infection (*n* = 5), ** *p* < 0.01; (**d**) *S. pneumoniae* in the lungs on 18 h after infection.

After viral-bacterial infection, L3-NA immunization protected 80% of mice from lethal influenza infection complicated by pneumococcal superinfection. The L3-NA immunized mice were 50% more protected from lethality in comparison with L3-immunized mice. (Figure 8a). In the PBS-vaccinated group, the survival rate was 40%, and in the L3-vaccinated group, the survival rate was even lower than in non-immunized mice, amounting to 30% (Figure 10a). The weight loss in mice immunized with L3-NA exceeding that in L3-immunized may indicate that more mice survived despite the severity of the infection (Figure 10b). Immunization with L3-NA reduced the viral load in the lungs, although it did not significantly affect *S. pneumoniae* levels in the lungs (Figure 10c,d).

## 4. Discussion

The aim of our study was to investigate the possibility of inserting a viral gene into the *E. faecium* L3 genome for expression of a viral protein in the pili. The choice of the minor antigenic component of the influenza virus, NA, for incorporation into *Enterococcus* genome was because NA is considered as a promising antigen for the development of broad-spectrum influenza vaccines [4]. In 2013, T. Doyle et al. [34] described one of the B cell epitopes in NA, conserved among all subtypes of influenza A. Monoclonal antibodies obtained against this epitope effectively inhibited in vitro the activity of influenza A viruses of all NA subtypes, and also protected mice against lethal infection. Conserved NA epitopes can be used in the design of a universal influenza vaccine [35,36], and also as an additional component to existing vaccines [37].

The idea of using bacteria by the oral route for vaccination has been proposed before. Several studies investigated the expression of viral, bacterial or eukaryotic origin proteins in probiotic microorganisms [38,39]. In 2015, Lei H. and colleagues created a chimeric construct based on the vector *Lactococcus lactis* (*L. lactis*) containing the NA of the influenza A virus on its surface, and showed that it was able to protect mice from infection with influenza viruses [2]. As in our study, it was shown that the detectable level of serum and local antibodies was formed after prime-boost immunization. The authors showed that such a chimeric construct could be a candidate for a universal influenza vaccine. 

Administration of immunogenic molecules through the mucous membranes has several important advantages over systemic administration. Mucosal vaccines are easy to administer, do not cause systemic reactions and they induce both systemic and local immune responses. The genetic fusion to the pili protein may improve of the antigenic presentation on a highly ordered and repetitive backbone that will enhance the immune response to the fused antigen. The pili of enterococci are very promising candidates for vaccine development due to their exposure to the cell surface. They protrude beyond the bacterial cells and are able to penetrate the capsule that screens most of the antigen proteins. Pili consist of monomers capable of aggregation, thereby increasing the dose of the antigens, which contributes to an increase in antibody titers specific to the antigens inserted into the structure of the surface protein of *Enterococcus*.

The development of a live vaccine against group B streptococcus (GBS) was described, using an approach based on the introduction of the BAC gene of pathogenic GBS into the chromosomal DNA of the probiotic strain *E. faecium* L3 when expressed in pili [40].

In our study, for the first time, it was shown the possibility of embedding of influenza NA immunogenic epitopes into pili of *E. faecium* L3. The success of this insertion was confirmed by studying the expression of the mRNA in modified bacteria. In addition, the specificity of the NA insert was shown in ELISA with human polyclonal sera containing NA-inhibiting antibodies. Immunoelectron microscopy showed that the attachment of polyclonal NA-specific polyclonal antibodies occurred along the entire length of the pili, which may indicate the multiple presence of the antigen on its surface. The presence of multiple pili on the bacterial surface allows supposing that the D2 portion of the fimbrial protein D2 (fimbrial isopeptide formation D2 domain protein) was sufficient for proper assembly of the pili. Pilot experiments were carried out on mice, which showed that oral administration of live probiotic vaccine based on modified *E. faecium* L3 elicited antibody response and protected animals from lethal infection with the pandemic influenza virus A/South Africa/3626/13(H1N1)pdm09.

The presence of antibodies to NA can moderate the course of infection, especially if the infection is caused by a strain with a new HA subtype [15]. Immunity to NA is considered to be more cross-reactive than strain-specific immunity to HA. Using virus-like particle technology, Smith et al. studied an experimental NA vaccine in a ferret model of A/H5N1 influenza infection; the vaccine was able to provide effective protection against lethal challenge with A/Indonesia/05/2005 virus [41]. For expression in the *E. faecium* L3 pili, we chose the NA region (AA 123–331) of N2, since according to the analysis data, it contains a number of conserved B cell epitopes. The ILRTQESEC and DNWKGSNRP epitopes were previously experimentally confirmed by other authors [31,34]. We tried to test whether an approach based on the insertion of conserved epitopes of the influenza virus into a bacterial vector can protect against heterologous influenza infection. Therefore, despite the fact that L3-NA contained neuraminidase of the N2 subtype, the challenge was with the pandemic influenza virus A/South Africa/3626/13(H1N1)pdm09. It was shown that L3-NA demonstrated 67% protection against heterologous influenza infection. Moreover, the developed mucosal vaccine candidate even protected 80% of mice against post-influenza pneumococcal pneumonia. The experiments with a mucosal vaccine against post-influenza *S. pneumonia* infection were carried out as the most common cause of severe outcomes after influenza are bacterial complications. In our earlier work, it was shown that pneumococcal infection alone was not fatal in mice, however, against the background of sublethal influenza infection, 100% mortality was observed [42]. Immunization with L3-NA significantly reduced mortality after sequential pneumococcal invasion following influenza infection. The fact that L3-NA immunization reduces the content of the virus in the lungs but does not affect the levels of pneumococci suggests that the reduction in mortality observed in this study was due to a reduction in influenza infection.

The data showed that L3 caused an increase in antibodies to influenza virus NA in mice, but did not protect, as L3-NA, suggesting that this protection was specific, which was also confirmed by the detection of NA-specific antibodies. Probiotic vector mucosal vaccines administered orally may be advantageous when frequent booster immunizations are needed, such as with COVID-19 [43]. Therefore, it is important to develop appropriate platforms, one of which is the insertion of viral epitopes into a bacterial vector *E. faecium* L3.

## 5. Conclusions

For the first time, the surface display of influenza virus NA was demonstrated in pili of the probiotic strain *Enterococcus faecium* L3.

Oral immunization of mice with a probiotic strain containing neuraminidase N2 subtype protected mice from lethality caused by pandemic influenza A/South Africa/3626/13 (H1N1)pdm09 influenza infection or post-influenza pneumococcal pneumonia. Such protection was associated with the formation of systemic and local virus-specific antibodies.

The approach based on a probiotic vaccine expressing viral epitopes can allow repeated vaccination during the epidemic season, even when conventional vaccination is contraindicated.

Oral administration of live probiotic vaccines generated by this technology may be promising for anti-viral protection of both humans and agricultural animals.

## Figures and Tables

**Table 1:** The influenza viruses used for the molecular analysis of the NA gene.

Subtype/Lineage	Name	Sequence Number
H2N2	A/Leningrad/134/17/1957	L37330
H2N2	A/duck/Pennsylvania/14-030488-005/2014	QHQ82622
H3N2	A/17/Singapore/2016/3571	EPI1313135
H3N2	A/South Dakota/12/2020	MT467157
H1N1	A/California/07/2009	HM138502
H1N1	A/South Africa/A201-004-041/2018	MN716456
H5N1	A/Viet Nam/1203/2004	ABP52008
H7N3	A/chicken/Karachi/NARC-100/2004	FJ577544
H7N9	A/Anhui/1-DEWH730/2013	CY187620
H7N9	A/Guangdong/HP001/2017	AQY18948
B/Victoria	B/Brisbane/60/2008	AFH57913
B/Yamagata	B/Wisconsin/04/2015	ALH38476

**Table 2:** Oligonucleotide primers.

Primers	Direction	Nucleotide Sequence from 5′ to 3′
EV	forward	GCATATGGCGATCCTGGCAAGTGTTAT ^1^
FV	reverse	GGAATTCCTAGAGCTGTCGTCGTTCC
A1	forward	GCTCTAGAGCCGATGAGAGCAGCTGGTATTG
D1	reverse	CAACAGGATCCAAAGCATCGTTGG
B1	forward	TGAGTGAACCACAGCCAGAA
Seq F	forward	GGACACCACAACCATCGAAG
Seq R	reverse	AGCTGGACCATGCTACACA

^1^ Underlined nucleotides indicate restriction sites.

## Supplementary Materials

The following are available online at https://www.mdpi.com/article/10.3390/microorganisms9122446/s1, Supplementary data: Nucleotide sequence showing integration of pentF-na plasmid DNA into *Enterococcus faecium* L3 chromosomal DNA.
