# Supplementary material for: Developing a Live Probiotic Vaccine Based on the Enterococcus faecium L3 Strain Expressing Influenza Neuraminidase
Source: Microorganisms. 2021 Nov 27;9(12):2446. doi: 10.3390/microorganisms9122446 (PMC8707194; doi:10.3390/microorganisms9122446)
Supplement: Supplementary file 1 [file microorganisms-09-02446-s001.zip › microorganisms-1408491-supplementary.pdf]

d2-1

TGAGTGAACCACAGCCAGAAATTAATTCAAAAATGAGATCGATGAGAGCAGCTGGT
ATTGAGTTGAATGATACATTTCTATCTATTTACAGTTTAAATGGACAGTATCAGCA
ACGTGTGTCTTGGTATAATGACAATAATGAATCTGTCGGTGAACGTAATATTGATA
TGAGAGAATTTGTTGGGTATGAAAAAATGGGTAGCTTACCTTATTTGTCACAACA
GATACAGCATGTGCAGAATACAAAGCTCCTGCGTTATCAACAAACAATTTAACTTC
AAAAGTAGTGGGAGGACGTGCAGAAAAGGCTTATAGCTCGAATGATCATTTCACCG
ATGTTGTAGGAGCTGATACTTATCACAGAAGTGGTGTAACGTATACGCTTCAAGGC
GCTTCCCCAACATTCATGATTGGCGCAAATACGAATAGTATGATGTTTAGCTTTGA
TACTGCATTGCTATGGACACCACAACCATCGAAGCCTACAAAAGAAGTGTTTAACA
AAGCTAATACTGAAGAGGCAGCACACAATATTGACAAAAAAGTGATTCCACAAGGA
TCAGATGTTTACTATCATATTCATCAAAAGTTTGATGCATTAACAGTCAACACAAT
GAACAAATACAAATCATTTAAAATCACTGATACCTTTGACAGCAAAAATTTTGATA
na
TGGTATCGGATGGGAAAAACTATGATGGCGCATTGCATATGGCGATCCTGGCAAGT
GTTATCAATGCGATCCTGGCAAGTGTTATCAATTTGCACTCGGGCAGGGGACCACA
CTAGACAACAAACATTCAAATGGCACAATACATGATAGAATCCCTCATCGAACCCT
ATTAATGAATGAGTTGGGTGTTCCATTTCATTTAGGAACCAAACAAG**TGTGTGTAG**
CATGGTCCAGCTC

Supplementary data S1. Nucleotide sequence showing integration of pentF-na plasmid DNA into *Enterococcus faecium L3* chromosomal DNA. The sequences of primers B1 and SeqR are highlighted in bold.
